# A role for *Egfl7 *during endothelial organization in the embryoid body model system

**DOI:** 10.1186/2040-2384-2-4

**Published:** 2010-02-19

**Authors:** Anna Durrans, Heidi Stuhlmann

**Affiliations:** 1 Department of Cell and Developmental Biology, Weill Medical College of Cornell University, 1300 York Avenue, New York, NY, 10065 USA

## Abstract

Epidermal growth factor-like domain 7, *Egfl7*, is a largely endothelial restricted gene which is thought to have a role during the differentiation of embryonic stem cells (ESCs) along the endothelial lineage. While it has been shown that *Egfl7 *knock-down in zebrafish impairs endothelial cord formation, the role of the gene in mammals has been unresolved. Interpretation of mouse knockout studies has been complicated by the fact that deletion of *miR-126*, an intronic microRNA located within *Egfl7*, results in vascular defects. Here we use an siRNA knock-down approach to target specific regions of *Egfl7 *without affecting *miR-126 *expression. *Egfl7 *was knocked down in mouse ESCs and the effect on vascular development was assessed using the *in vitro *embryoid body (EB) model after either 7 or 14 days of differentiation. Knock-down of *Egfl7 *resulted in the formation of abnormal sheet-like CD31+ structures that were abundant within EBs after 7 days of differentiation. Only up to 60% of these sheets co-expressed basement membrane and endothelial cell junction markers. Similar CD31+ sheets were also seen as outgrowths from 7 day EBs into collagen gels. A partial remodelling occurred by 14 days of differentiation when fewer CD31+ sheets were seen both within EBs, and as outgrowths from EBs. Formation of these sheets was due, at least in part, to increased proliferation specifically of CD31+ cells. Cell death within EBs was unaffected by *Egfl7 *knock-down. In conclusion, our work shows that knock-down of *Egfl7 *causes defects in early vascular cord formation, and results in the development of CD31+ sheet-like structures. This suggests that *Egfl7 *is vital for the formation of endothelial cell cords, and that the gene has an important role during both vasculogenesis and angiogenesis in mammalian cells.

## Background

Epidermal growth factor-like domain 7, *Egfl7*, was identified in a screen for genes with restricted expression during *in vitro *differentiation and mouse embryogenesis [1]. EGFL7 is expressed in undifferentiated mouse embryonic stem cells (ESCs), during early embryogenesis at sites of blood island formation and vasculogenesis, and in adults during pathological and physiological angiogenesis [1,2] (L. Campagnolo and H. Stuhlmann, Unpublished). Expression of EGFL7 is largely restricted to endothelial cells (ECs), and is down-regulated in most adult organs with the exception of the pregnant uterus and during wound repair [1,2]. In addition, expression has also been reported in primordial germ cells and male germ cells [3]. Due to its early and restricted expression, *Egfl7 *has been proposed to have a role during the differentiation of ESCs along the endothelial lineage. EGFL7 is a secreted protein which stimulates EC migration, and knock-down of the gene in zebrafish results in a severe impairment of arterial and venous EC cord segregation leading to the formation of midline angioblast aggregates [2,4,5]. However, the function of *Egfl7 *in mammalian vascular development is still unresolved. In a mouse knockout study, Schmidt *et al *[6] showed partial embryonic lethality, delayed vascular development, and abnormal EC aggregates. In contrast, Kuhnert *et al *[7] found no phenotype in *Egfl7 *knockout mice, and instead proposed that the observed vascular defects could be attributed to deletion of *miR-126*, an endothelial microRNA located within intron 7 of *Egfl7*. In this study we have used an siRNA knock-down approach, enabling us to target regions of the *Egfl7 *gene other than intron 7. This has allowed us to specifically investigate the role of *Egfl7 *during vascular development, without affecting *miR-126 *expression.

We chose the embryoid body (EB) differentiation model to examine the effect of *Egfl7 *knock-down on vasculogenesis and angiogenic sprouting. Numerous studies have shown that EBs facilitate the interaction of cells of the ectodermal, mesodermal, and endodermal lineages, recapitulating the developmental kinetics of normal mouse embryonic development [8-11]. Because EBs are initially formed by differentiating ESCs, this system allows for assessment of vascular structure development and the process of ESC differentiation to be assessed. Recent work has shown that vascular structures within EBs are surrounded by a basement membrane, as is the case for blood vessels *in vivo *[12]. We used EBs that were differentiated for 7 days, roughly equivalent to an early organogenesis stage, and 14 days, which is considered to be a later remodeling stage [13]. We also looked at the effect of *Egfl7 *knock-down on sprouting angiogenesis using EBs in a type I collagen gel. Here we show that *Egfl7 *knock-down results in the formation of abnormal endothelial sheet-like structures, which form during the initial stages of *in vitro *vascular development. During the subsequent processes of differentiation, presumably involving remodelling, endothelial cords replace a large proportion of these sheets. Our results suggest a role for *Egfl7 *in EC organization, and indicate that the gene is necessary for normal vascular growth during both vasculogenesis and angiogenesis in mammalian cells.

## Methods

### Knock-down construct and siRNA production

The siRNA sequences used for *Egfl7 *knock-down (KD1, KD2, KD3), and the scrambled controls (Scr1, Scr2) were as follows; KD1: 5'-UACUUGCCAGACAGAUGUU-3' (sense), 3'-UUAUGAACGGUCUGUCUAC-5' (antisense); KD2: 5'-GCAGCUGGACCGAAUUGAU-3' (sense), 3'-UUCGUCGACCUGGCUUAAC-5' (antisense); KD3: 5'-GCUCCCUGUCUAAGUGGUAA-3' (sense), 3'-UUCGAGGGACAGAUUCACCA-5' (antisense); Scr1: 5'-GCUCCCUAGGCUAGUGGUAA-3' (sense), 3'-UUCGAGGGAUCCGAUCACCA-3' (antisense); Scr2: 5'-UACUUGGACGACAGAUGUU-3' (sense), 3'-UUAUGAACCUGCUGUCUAC-5' (antisense). Sense and antisense oligonucleotides were annealed and ligated to linearised psiRNA-hH1neoG2 vector (Invitrogen) before sub-cloning into the FG12 lentiviral vector carrying an eGFP reporter sequence [14].

### Production of lentivirus and stable embryonic stem cell knock-down clones

HEK 293T cells were co-transfected with FG12 lentiviral vectors carrying the siRNA sequence, HIV-1 lentiviral packaging constructs (pMDLg/pRRE and pRSV-REV), and pVSV-G (a plasmid coding for the G protein of the vesicular stomatitis virus) by the calcium phosphate method. Virus supernatant was collected 24-40 h after transfection and concentrated by ultracentrifugation (22,000 × g). The virus titers were determined on 3T3 cells by counting the number of eGFP+ cells under a microscope, and were 5 × 10^6^-1.5 × 10^8 ^infectious units/ml. Mouse ESCs (W4/129S6; Taconic) were grown on a feeder layer of irradiated mouse embryonic fibroblasts (MEFs) in DMEM supplemented with 15% FBS, 20 mM HEPES, 0.1 mM non-essential amino acids, 0.1 mM β-mercaptoethanol, 100 U/ml penicillin/streptomycin, 0.3 mg/ml L-glutamine, and 10^3^U/ml LIF (ESGRO; Chemicon). ESCs were infected in the presence of 8 μg/ml polybrene at a MOI of 1-2. Individual eGFP+ clones were isolated and assessed for expression of the knock-down construct by RT-PCR and western blot. RNA and protein was extracted from cells using the PARIS kit (Ambion) as per manufacturer's instructions. Reverse transcription was carried out using the SuperScript III First-Strand Synthesis System (Invitrogen), followed by PCR using the following primers; *Egfl7*: 5'-ACAGACCCAGCCGTAGAGTG-3' (forward, spanning exons 3 and 4), 5'-TCAATTCGGTCCAGCTGCTGG-3' (reverse, within exon 9); *GAPDH*: 5'-ACCACAGTCCATGCCATCAC-3' (forward), 5'-TCCACCACCCTGTTGCTGTA-3' (reverse). For westerns, 40 μg protein was run on a 10% bis-tris gel (Invitrogen) under reducing conditions, and transferred to a PVDF membrane which was incubated with antibodies against EGFL7 [1] and actin (Santa Cruz), followed by HRP-conjugated secondary antibodies. Images of eGFP+ ESCs were taken with a Leica DFC340FX digital colour camera mounted on a Leica DMIL inverted microscope, using Leica Application Suite Software (Leica Microsystems), and eGFP+ EBs were visualized using a stereo Discovery. V20 microscope (Carl Zeiss) with an X-Cite 120 external fluorescent light source (EXFO Photonic Solutions Inc.)

### Real-time PCR analysis of *Egfl7 *and *miR-126 *levels

For real-time PCR analysis, total RNA was isolated from ESCs using the RNAqueous-Micro Kit (Ambion) as per manufacturer's instructions. *Egfl7 *levels were determined by carrying out reverse transcription as described above, followed by PCR using the following primers; *Egfl7 (*spanning intron 8) 5'-AGAGGAGGTGTACAGGCTGCA-3' (forward), 5'-TTCGGTCCAGCTGCTGGAAGGAAT-3' (reverse); *β-actin*: 5'-CCATCATGAAGTGTGACGTTG-3' (forward), 5'-CAATGATCTTGATCTTCATGGTG-3' (reverse). Levels of the microRNAs *miR-126-3p *and *miR-126-5p *were determined by first carrying out reverse transcription using microRNA-specific primers and the Taqman MicroRNA Reverse Transcription Kit (Applied Biosystems). PCR was then done using Taqman MicroRNA Assay (Applied Biosystems), and levels of expression were normalized to *miR-16*.

### Embryonic stem cell growth rate

ESC growth rates were determined essentially as described by Udy *et al *[15]. ESCs were plated on MEFs in 12 well plates at 30 cells/well. At each time point (4-8 days after plating) ESCs from triplicate wells were trypsinized, MEF-depleted, and counted using a haemocytometer. Averages of triplicate counts were compared by two-way ANOVA with repeated measures and a Bonferroni post-test using Prism4 (GraphPad Software, Inc.).

### Embryonic stem cell differentiation as embryoid bodies

ESCs were MEF-depleted and seeded in 30 μl hanging drops at 8 × 10^4 ^cells/ml in differentiation medium (as for ES cell media, except 20% FBS and no LIF). Two days later EBs were grown in suspension, and then harvested at either 7 or 14 days after initial seeding. Where TGF-β was used, 2.5 ng/ml recombinant human TGF-β (R&D Systems) was added to medium prior to making hanging drops, and also for subsequent feeding. In other experiments, conditioned medium from wild-type, or scrambled control, EBs was used on knock-down clones during differentiation to day 7 EBs. EBs were fixed in 4% PFA followed by 10% and 20% sucrose, and frozen in a 1:1 solution of OCT: 30% sucrose. 12 μm sections were used for indirect immunofluorescence (IF) staining.

### Collagen type I sprouting angiogenesis assay

Ten individual 7 or 14 day EBs were plated onto 1.5 ml of solidified collagen type I medium in a 35 mm diameter plate and allowed to settle overnight in differentiation medium, before a second collagen layer was added. The collagen medium was made as described by Feraud *et al *[16] with a final concentration of rat tail type I collagen of 1.25 mg/ml (BD Biosciences). Recombinant growth factors were used at final concentrations known to provide maximal biological stimulation: human VEGF_165_, 50 ng/ml; mouse FGF basic, 100 ng/ml; mouse Epo, 20 ng/ml; human IL-6, 10 ng/ml (R&D Systems) [17]. After nine days the gels were quickly dehydrated on glass slides using nylon linen and filter paper, and air-dried overnight before being stored at -80°C until staining.

### Indirect immunofluorescence staining

EB cryosections were fixed in ice-cold acetone, or methanol (for Flk1 staining), and EBs within collagen type I gels were fixed in 4% PFA. Samples were blocked with 10% normal donkey serum and 5% non-fat dried milk, and antibodies were diluted in 5% non-fat dried milk. Collagen gels were also permeabilized using 0.5% triton X-100. For Annexin-V staining 2% BSA was used instead of milk. Sequential double-staining was carried out with the anti-CD31 antibody first, and antibodies were used as follows; rat anti-mouse CD31, 5 μg/ml (BD Biosciences), goat anti-mouse Flk1, 4 μg/ml (Santa Cruz), rabbit anti-mouse Collagen IV, 5 μg/ml (Chemicon), goat anti-mouse VE-Cadherin, 5 μg/ml (R&D Systems), rabbit anti-mouse Claudin-5, 2.5 μg/ml (Invitrogen), rabbit anti-mouse Ki67, 1.5 μg/ml (Abcam), rabbit anti-mouse Annexin-V, 2.5 μg/ml (Abcam). Rat, rabbit, and goat IgG controls were used on adjacent sections. Signals were detected with donkey anti-rat IgG conjugated with Cy3 or Cy5, and donkey anti-rabbit or -goat IgG conjugated with Cy5 or Cy3 (Jackson ImmunoResearch). Cryosections were mounted in ProLong Gold Antifade reagent with DAPI (Invitrogen). Collagen gels were incubated with Hoechst 33342 nuclear dye (Invitrogen) and mounted in Vectashield hard-set mounting medium (Vector Labs). Images were taken using an Axioplan 2 imaging microscope (Carl Zeiss), or a Leica TSC SP2 confocal laser microscope (Leica Microsystems).

### Quantification of vascular structures

For quantification of EB cryosections and collagen gel-embedded EBs, image acquisition was performed with an ORCA-ER black and white camera (Hamamatsu Photonics) driven by Openlab software (Improvision, Ltd.). Relative CD31+, Ki67+, and DAPI+, areas were measured by determining the number of pixels corresponding to the fluorescent signal using the 'magic wand tool' in Photoshop (Adobe Systems Inc.). Individual EBs were also scored for the presence of CD31+ 'cords', 'sheets', or both. Cords were defined as CD31+ structures of more than two cells in length, and not more than two cells in width, determined by counting DAPI-stained nuclei in overlaid images. CD31+ sheet structures were defined as being more than four cells in diameter, and more than four cell's distance from another sheet to be counted individually. CD31+ sheets were also analyzed separately, and the Ki67+ pixels within each sheet determined. Where indicated, confocal images were captured using Leica Confocal Software. All analysis for the collagen gel-embedded EBs was done using Photoshop. Relative CD31+ sprout length was determined using the 'measure' tool and branching points were defined as where two or more CD31+ sprouts radiated from. All statistical analysis was carried out using Prism4 (GraphPad Software, Inc.).

## Results and Discussion

### Lentiviral-mediated knock-down of *Egfl7*

**Figure 1:**
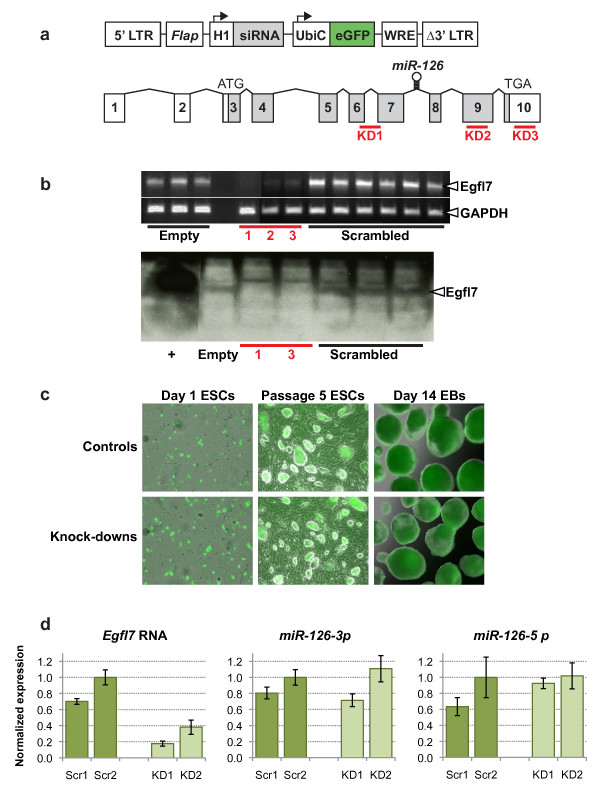
**Knock-down of *Egfl7 *expression in mouse embryonic stem cells**. (**a**) Lentiviral construct used for siRNA-mediated knock-down of *Egfl7 *(top), and *Egfl7 *gene structure (bottom) showing non-coding (unshaded) and coding (shaded) exons. The three siRNA target sequences are shown as red bars, and the location of the microRNA *miR-126 *is shown within intron 7. (LTR, long terminal repeat; *Flap*, DNA flap; H1, human H1 RNA pol III promoter; UbiC, human ubiquitin c promoter; eGFP, enhanced green fluorescent protein; WRE, woodchuck response element). (**b**) Verification of *Egfl7 *knock-down in mouse ESCs by RT-PCR (top) and western blot (bottom). (Empty, lentiviral construct alone; 1, 2, 3 (corresponding to KD1, KD2, KD3), lentivirus containing siRNA sequence; scrambled, lentivirus containing scrambled siRNA control sequence; +, HEK293 cells transfected with a His-tagged *Egfl7 *vector). (**c**) Endogenous eGFP expression in lentivirus infected undifferentiated ESCs 8 hours after plating (left two panels) and after passaging 5 times (middle two panels), and in ESC-derived EBs after 14 days of differentiation (right two panels). Magnification used 69×. (**d**) Quantitative PCR was carried out for *Egfl7 *and the microRNAs *miR-126-3p *and *miR-126-5p*. Expression was normalized to *β-actin *(for *Egfl7*) or *miR-16 *(for the microRNAs), and is shown relative to a value of 1.0 for the scrambled control 2 (Scr2).

The FG12 lentiviral vector [14,18] was used for delivery of siRNAs into ESCs. This vector has an RNA polymerase III promoter (H1) to drive siRNA expression, and an UbiC promoter to drive marker gene (eGFP) expression (Figure 1a). The use of RNA polymerase III and UbiC promoters is an established technique in lentiviral-mediated knock-down [14,19]. The eGFP expression serves as a reliable marker to demonstrate the presence and expression of the *Egfl7 *siRNA knock-down sequence. The siRNA constructs were designed to knock-down *Egfl7 *by targeting different regions of the *Egfl7 *gene; spanning coding exons 6 and 7 (KD1), within exon 9 (KD2), and in the 3-prime untranslated region in exon 10 (KD3) (Figure 1a). These constructs reduced *Egfl7 *expression at the RNA level by 75-95%, and also significantly reduced expression at the protein level (Figure 1b). Reporter eGFP expression was visible in stably infected ES cells, and for at least 14 days after differentiation *in vitro *(Figure 1c). Three control constructs were also used; one being the lentiviral vector with no siRNA sequence (Empty), and two containing scrambled sequences (Scr1, Scr2). ESCs were infected at an MOI of 1-2 providing a single or low proviral copy number per cell. Throughout all experiments results were consistent between the knock-down constructs, and between the empty and scrambled controls. Importantly, quantitative PCR showed that *Egfl7 *knock-down did not affect levels of the microRNAs *miR-126-3p *(3 prime end) or *miR-126-5p *(5 prime end) (Figure 1d), which are generated as a stem loop encoded by intron 7 within the *Egfl7 *gene (Figure 1a). We were therefore able to study the specific effect of *Egfl7 *knock-down, without any possible effects on altered *miR-126 *levels. Because Egfl7 expression is restricted to undifferentiated ES cells, early mesodermal precursors of vascular endothelial cells, and to the vasculature during development [1], lentiviral infection of ESCs will only affect the corresponding cell types in the differentiating EBs that normally express Egfl7.

### *Egfl7 *knock-down reduces embryonic stem cell growth rate

**Figure 2:**
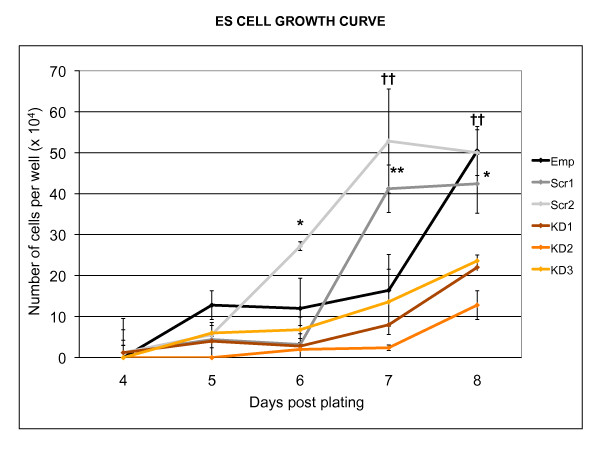
**Effect of *Egfl7 *knock-down on embryonic stem cell growth rate**. Knock-down of *Egfl7 *resulted in a reduced growth rate in undifferentiated mouse ESCs. The experiment was carried out three times, yielding similar results. (* p < 0.05, ** p < 0.01, †† p < 0.001; Emp, empty lentiviral construct; Scr1, Scr2, Scrambled siRNA sequences; KD1, KD2, KD3, Knock-down siRNA sequences).

To determine whether *Egfl7 *knock-down affected undifferentiated ESC proliferation rate, cell counts were recorded between days 4 and 8 after plating at low density. Knockdown was associated with a significantly reduced growth rate compared with those infected with empty lentivirus and scrambled controls (Figure 2). *Egfl7 *is expressed at the RNA and protein level in undifferentiated ESCs [1], (L. Campagnolo and H. Stuhlmann, Unpublished data), and therefore this result suggests that *Egfl7 *has a role in ESC proliferation or self-renewal.

### Abnormal endothelial sheets form in *Egfl7 *knock-down embryoid bodies

**Figure 3:**
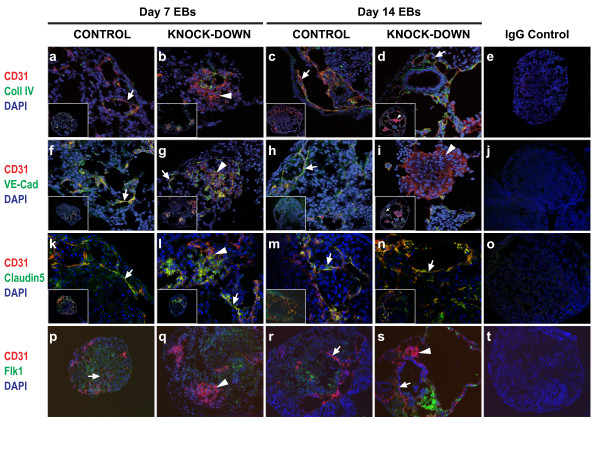
**Effect of *Egfl7 *knock-down on *in *vitro endothelial development**. Cryosections of EBs at day 7 (a, b, f, g, k, l, p, q) and day 14 (c, d, h, i, m, n, r, s) were subjected to indirect IF using antibodies against CD31 plus collagen IV (a-d), CD31 plus VE-cadherin (f-i), CD31 plus claudin 5 (k-n), or CD31 plus Flk1 (p-s). Magnification used; (a-d, f-i, k-n) inserts show whole EBs at 20×, and large panels show detail at 63×, (p-s) panels show EBs at 20×, (e, j, o, t) panels show whole EB IgG controls at 20× (arrows, CD31+ cords; arrowheads, CD31+ sheets). Images were acquired using a confocal laser microscope (Leica Microsystems; a-o) or an Axioplan 2 imaging microscope (Carl Zeiss; p-t).

EBs were formed from the three knock-down and three control ESC clones by the hanging drop method [20]. *In vitro *differentiation occurred at similar rates for all clones, with no differences observed in the size or number of EBs (data not shown). During *in vitro *differentiation EBs grow from simple aggregates of ES cells at 2-4 days, to being cystic EBs which resemble the visceral yolk sac of post-implantation embryos after 8-10 days [8,13]. In the present work this progression was observed between the earlier (7 day) and later (14 day) time points, and cysts/cavities can clearly be seen in cryosections of day 14 EBs (e.g. Figures 3c and 3d). Normal developmental processes account for the formation of cavities, as well as complex networks of cords, within the day 14 EBs. Furthermore, Annexin V staining did not indicate abnormally high levels of apoptosis/necrosis in the day 14 EBs, thus indicating that there was no increased degradation associated with cavitation (see below).

Vascular structures within EBs were analyzed at 7 days and 14 days of differentiation, as these time points correspond approximately with early organogenesis and later remodelling stages respectively [13]. Sections through EBs revealed the presence of two clearly distinguishable CD31+ cell structures, which we describe here as 'cords' (Figure 3; arrows) and 'sheets' (Figure 3; arrowheads). 'Cords' are defined as more than two CD31+ cells in length and a maximum of two CD31+ cells in width, and 'sheets' as aggregates of more than four CD31+ cells in length and width. These structures were viewed on 2-dimensional cryosections through EBs. Due to the spherical shape of EBs it is probable that the 'cords' are part of a larger network existing in multiple axes within the EBs, and that the 'sheets' represent one plane within 3-dimensional clusters of CD31+ cells. Similar CD31+ 'sheets' have been described in EBs derived from laminin γ1-deficient ESCs [12]. To further characterize the CD31+ structures present, EB sections were co-stained with antibodies against other endothelial markers. Collagen IV is a major constituent of the basement membrane, and its deposition is characteristic of normal blood vessel formation and is required for subsequent angiogenesis [12,21,22]. Vascular endothelial (VE)-cadherin is the major transmembrane component of adherens junctions, and sustains cell-cell recognition and adhesion [23]. Used together with CD31, which is expressed on haematopoietic cells as well as ECs, VE-cadherin is considered to be the gold standard for EC-specific markers [24]. Claudin-5 is an endothelial-specific component of tight-junctions, which control para-cellular permeability and polarity [25,26]. Flk1, a VEGF-A receptor, is an early marker of hematopoietic and endothelial cells [27].

**Figure 4:**
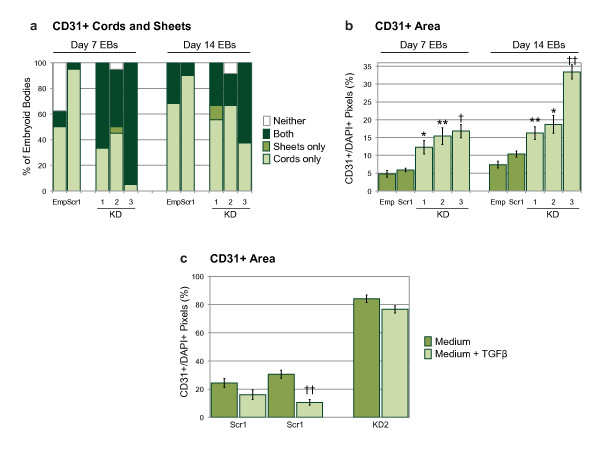
**Quantification of CD31+ structures within embryoid bodies**. **(a) **Percentage of EBs containing CD31+ cords only, sheets only, both cords and sheets, or neither structure, as defined in Methods. **(b) **Relative CD31+ area on EB sections, measured in pixels and shown as a ratio of DAPI+ pixel number. Statistical significance was determined by one-way ANOVA followed by a Bonferroni post-test. **(c) **Relative CD31+ area on EBs sections; EBs grown +/- TGF-β. Statistical significance was determined by two-tailed, unpaired, t-tests. Bars are means ± S.E.M. (Emp, empty lentiviral construct; Scr1, scrambled siRNA sequence; KD1-3, knock-down siRNA sequences; *n *= 16-36 for controls, *n *= 20-29 for knock-downs; * p < 0.05, ** p < 0.01, † p < 0.005, †† p < 0.001). The data shows results from one experiment, which was carried out twice with similar results.

At the day 7 time point CD31+ cords which co-expressed collagen IV, VE-cadherin, and claudin 5 were seen in all of the control and knock-down clones (Figures 3a, f, g, k, and 3l; arrows). However, the presence of large aggregates of CD31+ cells clearly distinguished the knock-down clones from the controls (Figures 3b, g, l, and 3q; arrowheads). By day 14 of differentiation more extensive endothelial cord networks had formed in all of the clones (Figures 3c, d, h, i (inset), 3m, n, r, and 3s; arrows), and some sheets were still present in the knock-down EBs (Figures 3d (inset), 3i, and 3s). Quantification of the CD31+ structures revealed a striking difference between controls and knock-downs. At day 7 the majority of the control EBs contained cords, whereas most of the knock-downs contained sheets as well as cords (Figure 4a). The spatial organization of ECs within these sheets were reminiscent of the midline angioblast aggregates seen in *Egfl7 *knockdown zebrafish, as well as the sheet-like endothelial projections seen in aortic ring assays using *Egfl7 *knock-out mice [4,6]. In the EB model CD31+ sheets were seen at the early organogenesis stage and less so at the later remodelling time point, suggesting a role for *Egfl7 *specifically at early stages of cord-formation, and subsequent remodelling of some of these structures. The percentage of CD31+ sheets in the knock-down clones which co-expressed other endothelial proteins fell from 15% (collagen IV), 58% (VE-cadherin), and 60% (claudin 5) at day 7, to 7% (collagen IV), 31% (VE-cadherin), and 43% (claudin 5) at day 14. Furthermore, Flk1 did not co-localize with the CD31+ sheets (Figures 3q and 3s). Because expression of Egfl7 during ESC differentiation precedes that of Flk1 [1], Egfl7 knock-down may affect very early stage endothelial progenitor cells, suggesting that the CD31+ sheets are unlikely to contain immature endothelial cells. Together, these data indicate that the CD31+ sheets are aggregates of abnormal, vascular endothelial structures, which do not lay down an extensive basement membrane, or form normal cell-cell junctions. To determine whether knock-down of *Egfl7 *affected the size of CD31+ areas within EB sections, pixel numbers were compared between clones. Using Photoshop software (Adobe Systems Inc.) the number of CD31+ pixels on images of EB sections was normalized to the number of DAPI+ pixels, to account for variation in EB size. *Egfl7 *knock-down clones had a larger relative CD31+ area compared with controls at both the 7 and 14 day time points (Figure 4b), which is most likely to be due to the presence of sheets in the knock-down EBs. At the 14 day time point the number of sheets within the knock-down clones was decreased (Figure 4a), suggesting that the more extensive cord networks accounted for the larger CD31+ area, compared with the controls. At the early time point CD31+ sheets were far less prevalent in control EBs than in *Egfl7 *knock-down clones, indicating that these structures are not part of a normal vascular development process. Knock-down of *Egfl7 *resulted in fewer EBs containing CD31+ cords only, and a higher percentage containing both cords and sheets (Figure 4a). It is possible that at the later time point sprouts may form from cells contained within sheets, or that sheets form at the expense of, or in addition to, cords. However, it is most likely that sheets form in addition to cords, since few EBs contain sheets only.

When conditioned medium from wild-type, or scrambled control, EBs was added to cultures of knock-down ESCs during differentiation to day 7 EBs, we did not observe a rescue of the mutant phenotype (data not shown). This suggests that Egfl7 may act in a cell-autonomous manner. It has recently been shown that inhibition of the anti-proliferative transforming growth factor beta (TGF-β) during *in vitro *differentiation of human ESCs results in a 36-fold increase in the number of committed ECs generated [28]. Therefore, to address whether EGFL7 and TGF-β might interact, EBs were grown in the presence of TGF-β, which resulted in no significant decrease in CD31+ area in knock-down EBs, whereas control EBs showed a more robust decrease (Figure 4c). Thus, if TGF-β is involved in the function of Egfl7, it is unlikely to have a major role in maintaining the CD31+ cell population.

### CD31+ sheets show increased cell proliferation

**Figure 5:**
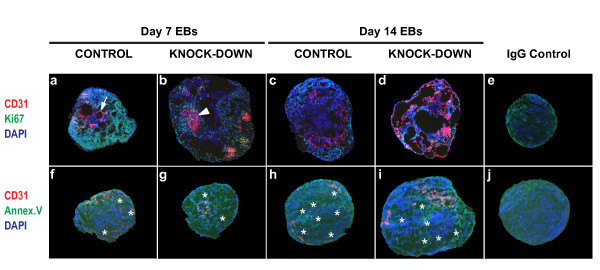
**Effect of *Egfl7 *knock-down on embryoid body proliferation and apoptosis**. Cryosections of EBs differentiated for 7 or 14 days were subjected to indirect IF using antibodies against CD31 plus Ki67 (40× magnification used; upper panels, a-d), or CD31 plus Annexin V (20× magnification used; bottom panels, f-i), (arrow, CD31+ cord; arrowhead, CD31+ sheet; asterisks, Annexin V+ cells). IgG controls are shown at 40× and 20× magnification (e, j). Images were acquired using an Axioplan 2 imaging microscope (Carl Zeiss).

**Figure 6:**
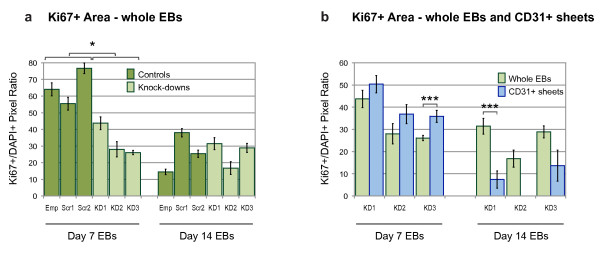
**Quantification of Ki67+ cells within embryoid bodies**. The effect of *Egfl7 *knock-down on *in vitro *proliferation was quantified in day 7 and day 14 EBs using an antibody against Ki67. Relative Ki67+ area was measured in pixels and shown as a ratio of DAPI+ pixel number. **(a) **Whole EB sections were compared between control and knock-down clones at each time point (*n *= 10-16 for controls, *n *= 9-23 for knock-downs). **(b) **Relative Ki67+ area of whole EBs, and CD31+ sheet areas, in knock-down EB clones. Statistical significance was determined by two-tailed, unpaired, t-tests. Bars are means ± S.E.M (Emp, empty lentiviral construct; Scr1, Scr2, scrambled siRNA sequences; KD1-3, knock-down siRNA sequences; *n *= 9-23 for whole EBs, *n *= 1-47 for CD31+ sheets; * p < 0.05, ** p < 0.01, † p < 0.005, †† p < 0.001).

To address whether over-proliferation or reduced apoptosis of CD31+ cells contributed to the formation of the abnormal aggregates in knock-down clones, EB sections were co-stained with antibodies against Ki67 and Annexin V. The nuclear protein Ki67 is a marker for proliferation, and Annexin V is visible only in apoptotic and necrotic cells [29,30]. At the 7 day time point there were many Ki67+ cells in the control EBs, whereas the knock-downs contained fewer of these cells, indicating reduced proliferation compared with the controls (Figures 5a and 5b; Figure 6a). At the 14 day time point all of the EBs, regardless of genotype, contained fewer Ki67+ cells compared with the 7 day EBs (Figures 5c and 5d; Figure 6a). In contrast, the number of cells expressing Annexin V increased over time, but did not differ between the controls and knock-downs (Figures 5f, g, h, and 5i). Despite a significant reduction in proliferation compared with controls (Figure 6a), all of the day 7 knock-down clones showed at least equal, if not higher, levels of proliferation within CD31+ sheets compared with the whole EB area (Figure 6b). This suggests that while knock-down of Egfl7 resulted in reduced proliferation, the abnormal CD31+ sheet areas proliferated as much as, if not more than, the rest of the EB structure. Since Egfl7 knock-down did not affect Annexin V levels (Figures 5f, g, h, i, and 5j), this suggests that the CD31+ sheets were not the result of localized cell death. It is therefore likely that these sheets occur, at least in part, due to over-proliferation of CD31+ cells in the knock-down clones. At the later time point the number of CD31+ sheets was markedly reduced, and those that existed contained a much lower proportion of proliferative cells than in the entire EB. Since knock-down of *Egfl7 *reduced both the proliferation of undifferentiated ESCs (Figure 2) and the overall proliferation in day 7 EBs (Figure 6a), CD31+ sheets are unlikely to be the result of a general increase in numbers of ESCs or cells within EBs.

### *Egfl7 *knock-down is associated with endothelial sheet formation during sprouting angiogenesis

**Figure 7:**
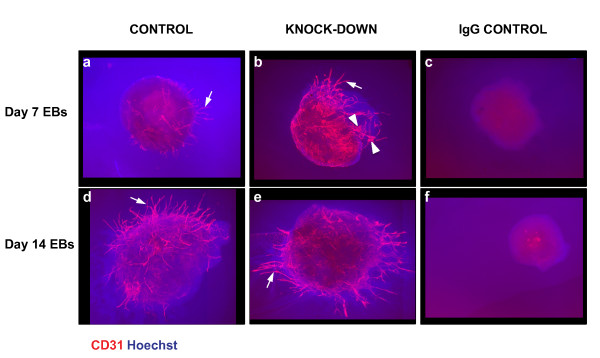
**Effect of *Egfl7 *knock-down on embryoid body sprouting angiogenesis**. Day 7 and day 14 differentiated EBs were grown between two layers of collagen type I gel for 11 days, before being subjected to indirect IF using an antibody against CD31, and Hoechst nuclear dye (arrows, CD31+ cords; arrowheads, CD31+ sheets).

**Figure 8:**
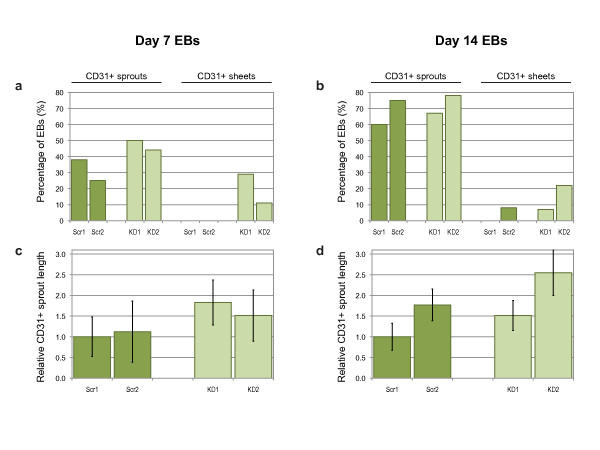
**Quantification of CD31+ sprouting angiogenesis**. Percentage of day 7 (**a**) or day 14 (**b**) EBs with either CD31+ sprouts or CD31+ sheets. Average CD31+ sprout length from day 7 (**c**) or day 14 (**d**) EBs, normalized to Scr1 values. Bars are means ± S.E.M. (Scr1 and Scr2, scrambled siRNA sequences; KD1 and KD2, knock-down siRNA sequences; *n *= 10 and 12 for controls, *n *= 15 and 9 for knockdowns).

We next examined the effect of *Egfl7 *knock-down on sprouting angiogenesis by seeding day 7 and day 14 EBs between layers of type I collagen and maintaining them for 11 days, a method which has been shown to recapitulate the early stages of angiogenesis [31]. Type I collagen is the major peri-capillary connective tissue protein and interacts with EC surface proteins during vessel sprouting and network formation [32], thus providing a physiologically relevant milieu for angiogenesis assays. EBs were differentiated as before for 7 or 14 days, and then placed between two layers of collagen gel in the presence of growth factors known to stimulate angiogenesis [17]. All EBs grew CD31+ sprouts (Figures 7a, b, d, and 7e; arrows) however *Egfl7 *knock-down clones also grew sheets of CD31+ cells (Figure 7b; arrowheads). Quantification of the CD31+ outgrowths from 7 day EBs showed that a higher percentage of knock-down clones contained sprouts compared with the controls, and that only knock-down clones showed sheet formation (Figure 8a). EBs placed in the collagen gel after 14 days of differentiation showed no differences in the number of EBs with sprouts or sheets (Figure 8b). Compared with the controls, day 7 knock-down EBs showed a 35-83% increase in relative sprout length, however this was not statistically significant due to high variation between EBs (Figure 8c). Day 14 knock-down EBs did not show consistent differences in CD31+ sprout length compared with the controls (Figure 8d). Control and knock-down EBs showed no differences in the number of sprouts per EB or the number of branch points, at either time point (data not shown). It is interesting to note that in the *Egfl7 *knock-down clones more CD31+ sheets grew from the EBs that had been differentiated for 7 days rather than 14 days, prior to placing in collagen. *Egfl7 *knockdown EBs differentiated for 7 days presumably contained CD31+ sheets prior to the sprouting assay (Figure 4). This suggests that early defects caused by knock-down of *Egfl7 *during development also affected EC sprouting during subsequent angiogenesis. Together, these results indicate that *Egfl7 *may be involved in sprouting angiogenesis, as knock-down of the gene resulted in a trend towards longer sprouts, as well as formation of sheets of CD31+ cells.

Recent work by Kuhnert *et al *[7] suggests that Egfl7-null mice are phenotypically normal, and that deletion of *miR-126 *causes embryonic lethality, edema, and hemorrhage, and postnatal defects in retinal and cranial angiogenesis. Thus, a possible role of Egfl7 in mammals has so far been elusive. Our studies are the first to show a clear role for Egfl7 in the formation of vascular structures in the EB *in vitro *differentiation model. In support, recent studies in transgenic mice show that Egfl7 overexpression results in hemorrhaging and defects in embryonic and post-natal angiogenesis (D. Nichol and H. Stuhlmann, Unpublished). The apparent discrepancy between studies using mouse knockout models, and the present work, could be explained by the fact that the Egfl7 phenotype detected in EBs is subtle, early, and transient. A strength of using the EB model system is the possibility to detect a transient and rather subtle phenotype in Egfl7 knock-down clones, which is evident at day 7 of differentiation, and then partially remodeled by day 14.

In conclusion, our results suggest that *Egfl7 *is vital for the organization of ECs into vascular cords and confirm that the gene has an important role during vasculogenesis and angiogenesis. We have shown that knock-down of *Egfl7 *results in the formation of CD31+ sheets, and our data support the notion that this is caused at least in part by the over-proliferation specifically of ECs during vasculogenesis. The CD31+ sheets appear to be abnormal endothelial structures lacking a complete basement membrane and cell junctions, which after further differentiation are accompanied by the formation of extensive endothelial cord networks. This indicates that partial remodelling occurs within the EBs, and points to an early developmental role for *Egfl7*. Thus, using an siRNA knock-down approach which did not affect *miR-126 *levels, we show here for the first time that *Egfl7 *has a role during endothelial cell differentiation and vascular development in mammalian cells.

## Competing interests

The authors declare that they have no competing interests.

## Authors' contributions

AD carried out the experimental studies and drafted the manuscript. AD and HS conceived of the study, and HS participated in its design and coordination, and helped to draft the manuscript. Both authors read and approved the final manuscript.
